# Diacutaneous Fibrolysis: An Update on Research into Musculoskeletal and Neural Clinical Entities

**DOI:** 10.3390/biomedicines11123122

**Published:** 2023-11-23

**Authors:** María Orosia Lucha-López, César Hidalgo-García, Sofía Monti-Ballano, Sergio Márquez-Gonzalvo, John Krauss, Héctor José Tricás-Vidal, José Miguel Tricás-Moreno

**Affiliations:** 1 Unidad de Investigación en Fisioterapia, Spin off Centro Clínico OMT-E Fisioterapia SLP, Universidad de Zaragoza, Domingo Miral s/n, 50009 Zaragoza, Spain; smonti@unizar.es (S.M.-B.); 724250@unizar.es (S.M.-G.); hjtricas@unizar.es (H.J.T.-V.); jmtricas@unizar.es (J.M.T.-M.); 2 School of Health Sciences, Oakland University, Rochester, MI 48309, USA; krauss@oakland.edu

**Keywords:** Diacutaneous Fibrolysis, connective tissue, musculoskeletal, neural

## Abstract

Diacutaneous Fibrolysis (DF) is an instrumentally assisted manual therapy technique defined as “a specific instrumental intervention for normalizing the musculoskeletal system function after a precise diagnosis and preserving the skin’s integrity”. The aim of this technique is soft tissue mobilization with the assistance of specially designed, hook-shaped steel instruments in different musculoskeletal structures, such as the myofascia, aponeurosis, tendons, ligaments and scar tissues. Due to discrepant results between previous reviews and the quite abundant new evidence provided by recently published randomized clinical trials, we propound this narrative review to provide an update on the scientific evidence related to the fundamentals and clinical efficacy of DF. Current evidence primarily supports the mechanical effect of DF on connective soft tissues. Diminished deep tendon reflex and rigidity have been registered after the implementation of DF in healthy subjects. Though there is still much to uncover, scientific evidence supports the use of the technique for the clinical treatment of subacromial impingement syndrome, chronic lateral epicondylalgia, chronic patellofemoral pain syndrome, mild to moderate carpal tunnel syndrome, hamstring shortening, temporomandibular disorders, tension-type headache and chronic low back pain. Additional data are essential for better recommendations in the clinical practice of DF.

## 1. Introduction

Diacutaneous Fibrolysis (DF) is an instrumentally assisted manual therapy technique which is defined as “a specific instrumental intervention for normalizing the musculoskeletal system function after a precise diagnosis and preserving the skin’s integrity” [1]. The application of this soft tissue mobilization is assisted by specially designed, hook-shaped steel instruments and applied to different musculoskeletal structures, such as the myofascia, aponeurosis, tendons, ligaments and scar tissues [1].

Based on deep transversal massage from Cyriax, DF was developed by Kurt Ekman, a Swedish physiotherapist, who assisted James Cyriax in the 1950s [2]. As a technique eminently based on the manual skills of the therapist who applies it, it has been transmitted to the present day thanks to the master–disciple model of knowledge transmission. The disciple of Kurt Ekman, Pierre Duby, a Belgian physiotherapist and professor of anatomy at the Free University of Brussels, spread the technique internationally, particularly in Europe. Most of the clinical research on DF has been carried out in Spain, where the technique was introduced by Pierre Duby in the 1990s at the University of Zaragoza. At the University of Zaragoza, José Miguel Tricás-Moreno and María Orosia Lucha López, two Spanish physiotherapists, started the diffusion of the technique in Spain.

The hook-shaped steel instruments are designed to promote a mechanical advantage by allowing deeper and more precise tissue mobilization and a reduction in stress on the fingers of the clinician [2]. Like deep transversal massage from Cyriax, DF is applied according to the tissue healing phase and transversal to the longitudinal direction of the tissue fibers to be treated [3], seeking mechanical, circulatory and neurological effects [4,5].

A systematic review and meta-analysis from 2021, including six randomized controlled trials performed to evaluate the effectiveness of DF on pain, range of motion and functionality in a variety of musculoskeletal disorders, stated the efficacy of the technique for pain relief and for the improvement of functionality immediately after treatment and at follow-ups of at least three weeks [6].

Moreover, there are some systematic reviews that have included DF as a potential co-adjuvant treatment for the management of a variety of musculoskeletal disorders.

Landesa-Piñeiro and Leirós-Rodríguez performed a systematic review about physiotherapy management in lateral epicondylitis [7]. Among the 19 manuscripts analyzed, one double-blind randomized controlled trial evaluating the effectiveness of DF for the treatment of chronic lateral epicondylalgia was included [8]. The authors stated that interventions including shock waves, platelet-rich plasma, ultrasounds, friction and stretching exercises, and bandages obtained better results with less sessions in pain relief. However, the dosage of the DF intervention that appears in the review is wrong, since it describes 15 sessions of DF [7] and the original manuscript specified that the dosage in the DF group was 6 sessions [8].

Haik et al. performed a systematic review of randomized controlled trials about the effectiveness of physical therapy in subacromial pain [9]. Of the 64 studies included, one double-blind randomized controlled trial evaluating the effectiveness of DF for the treatment of subacromial impingement syndrome was included [10]. We agree with the authors about their reflection stating that the effects of DF are not yet well established and that more studies are needed on the issue, but the review describes the comparing intervention in the control group of the DF study as an exercise intervention, and this is not exactly accurate. The control group of the DF study received five sessions per week of treatment that consisted of therapeutic exercises, analgesic electrotherapy and cryotherapy. The inclusion of analgesic electrotherapy and cryotherapy in the control group might have influenced the positive outcomes in pain achieved in the control group that Haik et al. attributed to supervised exercises alone [9].

Piper et al. performed a systematic review about the effectiveness of soft tissue therapy for the management of musculoskeletal disorders and injuries of the upper and lower extremities [11]. They analyzed six studies due to their low risk of bias, including the double-blind randomized controlled trial, evaluating the effectiveness of DF for the treatment of subacromial impingement syndrome [10]. Regarding DF, Piper et al. stated that “statistically significant differences favored DF, as well as sham DF over multimodal care for function, shoulder extension and external rotation” [11], but in the original manuscript, it was stated that “differences between placebo and control groups were statistically significant only in extension movement” [10], and the other differences were presented between DF and multimodal care.

Finally, Yu et al., in a review about the noninvasive management of soft tissue disorders of the shoulder [12], recommended that DF should not be offered for soft tissue disorders of the shoulder after analyzing the results of the previously mentioned double-blind randomized controlled trial [10]. Although the results were similar between the group treated with DF and the control group after three months, immediately after treatment, that is, after three weeks, including six DF sessions improved the functionality and the active extension and external rotation movements compared to the control group and the differences between the placebo and control groups were statistically significant only in extension movement.

Thus, due to the debatable results from some of the previous reviews and the quite abundant new evidence provided by the recently published randomized clinical trials, we propound this narrative review to provide an overview of the current evidence related to fundamentals and clinical efficacy of DF. We have hypothesized that due to the potential effect of DF in the remodeling process of the connective tissues after injury, intervention with DF in a variety of clinical entities, including connective tissue dysfunctions, may ameliorate the clinical course of these entities. Thus, the aim of this narrative review was to describe the potential mechanisms of action of DF and to assess the effectiveness of DF in the treatment of different musculoskeletal and neural clinical entities.

## 2. Materials and Methods

PubMed and Alcorze (search tool of the University of Zaragoza that simultaneously searches the main databases) were searched for randomized clinical trials, experimental studies and reviews up to 20 September 2023. Studies were included if they investigated the mechanisms of fibrinolysis or fibrolysis or massage in collagen and if they investigated the DF technique. Studies were excluded if they were single-case series or single-case reports, letters and editorials. No date or article language restriction was applied. Reference lists of selected articles were manually searched to identify other potential and relevant studies.

The search terms for the first search about the potential mechanisms of action of DF, in PubMed were (fibrinolysis OR fibrolysis) AND (collagen OR fascia) NOT (“hemo*” OR venous OR liver OR hepatic OR lung OR arteria) in which all capitalized words represent the Boolean operators used. A total of 339 results were obtained. After the revision of the title and/or the abstract, 2 articles were included.

A systematic search was also performed using the key terms Massage AND (collagen OR fibrolysis OR fibrinolysis). A total of 96 results were obtained. After the revision of the title and/or the abstract, 8 articles were included.

A systematic search about the effectiveness of DF in the treatment of different musculoskeletal and neural clinical entities was performed in PubMed. The search terms were fibrolysis AND “*cutaneous”. A total of 175 results were obtained. After the revision of the title and/or the abstract, 20 articles were included.

A systematic search was also performed in Alcorze using the key term Crochetagem. A total of 6 results were obtained. After the revision of the title and/or the abstract, 2 articles were included.

An additional 6 citations were included through the scanning of the reference lists of the previous studies.

## 3. Potential Mechanisms of Action of DF

DF, such as Cyriax’s massage, is supposed to accelerate and ameliorate the healing process in connective tissues, through mechanisms such as hyperemia with consequent increased blood flow to the tissue and elimination of the augmented cross-linking of collagen, as well as a decrease in the preponderance of disorganized type III collagen, and mechanoreceptor stimulation [13].

During the remodeling process of the connective tissues after injury, the proliferation of fibroblasts may lead to matrix accumulation by increasing the production of collagen type I and III, unbalanced with the degradation [14]. Moreover, fibroblast dysfunction has been related with hypoxia [15].

In a typical connective tissue disorder such as Dupuytren’s disease [16], fibroblasts proliferate and differentiate into myofibroblasts with an augmented contractile force. This force is beneficial for physiological tissue remodeling but it can become excessive, leading to connective tissue contracture with a subsequent deficit for function [17]. Tendinopathy, which is understood as maladaptation to mechanical loading, has been related to a degenerative process more than to an inflammatory one [13]. Tendon degeneration has been characterized by collagen fiber disorganization, disruption and angiogenesis [18]. In joint capsules of long-term immobilized joints suffering from hypomobility, increased disorganization of fibroblasts, high collagen density and irregular collagen fibers, as well as the disappearance of adipocytes in the synovial membrane, have been found [19].

Thus, due to these underlying mechanisms related to failed remodeling in the connective tissue, it might be supposed that a reduction in cross-linking and the disorganization of collagen and better blood flow will favor adequate tissue healing.

There are no studies in DF supporting the previous mechanisms nor the underlying mechanism behind them. Nevertheless, some studies developed in animal models have analyzed the tissue effects of other instrumentally assisted soft tissue mobilization approaches. They shed some light on the issue.

Kami et al. developed a study to highlight the effects of mechanical massage manipulation in the recovering of a blunt muscle injury in the gastrocnemius muscle of the rat. The blunt muscle injury was induced by a hit with 1.57 joules [20]. Massage was performed for 10 min per day for 25 days [14]. Collagenous fiber increased in the non-treated group with respect to the massage group. Muscle fibrosis is characterized by the accumulation of extracellular matrix, due to an imbalance between the synthesis and degradation of matrix components. An excessive number of fibroblasts and myofibroblasts can lead to extracellular matrix augmentation. In the massage group, the apoptosis of myofibroblasts was increased and showed a more orderly arrangement of sarcomeres within one myofibril [14].

Kassolik et al. studied the effects of the spiral friction technique, applied for 6 min per day, for 60 days, in the middle part of the tail of male rats, on the dense connective tissue of the tendon [21]. The diameter of collagen fibrils was smaller in the massage group with respect to the non-treated group. Tendon tissue in young rats is characterized by a smaller fibril diameter [21].

Andrzejewski et al. studied the effects of spiral friction movements on the collagen fibers of the rear long flexor muscle of the digits tendons from rats subjected to running training for 5 min per day, for 70 consecutive days [22]. In the same way as Kassolok et al., they found a higher percentage of the smallest fibers in the tendons [22].

Davidson et al. studied the effects of instrumentally assisted soft tissue mobilization in the Achilles tendons of rats with collagenase-induced tendinopathy [23]. The tendons received friction in a longitudinal manner from distal to proximal and from proximal to distal with an aluminum instrument for 3 min, four times, on post-collagenase injection days 21, 25, 29, and 33 [23]. In the tendons that received the soft tissue mobilization technique, more fibroblasts, stimulated extracellular matrix production and fibronectin (an extracellular matrix adhesion protein) were found [23]. Gehlsen et al., using the same methodology but with different pressure levels, discovered the largest number of fibroblasts after six sessions with the heaviest pressure [24].

Again using instrumentally assisted soft tissue mobilization in the collagenase-induced injured Achilles tendons of rabbits, Imai et al. found better dynamic viscoelasticity and a larger cross-sectional area in the treated tendons [18]. Collagen was better aligned in the treated tendons and a lower proportion of type III collagen was found, suggesting a better remodeling phase [18].

In 2009, Loghmani and Warden studied the effects of instrumentally assisted cross-fiber massage on the healing process of knee medial collateral ligament injuries [25]. The cross-fiber massage (transversal to the longitudinal direction of the ligament) was started 1 week post-injury and three sessions of 1 min per week were performed. Changes were evaluated at 4 and 12 weeks post-injury. At 4 weeks, the post-injury-treated ligaments had better orientation and formation of collagen fibers, with minimal differences at 12 weeks post-injury [25]. In 2013, the same authors, with a similar methodology, found enhanced tissue perfusion due to an increase in the proportion of arteriole-sized blood vessels in the tibial third of the ligament [26].

Subtle mechanical stretching consisting of 20 to 30% strain for 10 min, twice a day, for seven days, in mouse subcutaneous connective tissue of the back was related to a lesser presence of type-1 procollagen (in vivo) [27].

Some studies have been performed in healthy human subjects to study the effects of DF on the neural underlying mechanisms of muscular relaxation empirically observed with the technique.

Lévénez et al. found a reduction in passive tension, with augmentation of the maximal passive range of motion in ankle dorsiflexion and a reduction in the deep tendon reflex from the triceps surae, but not in the Hoffmann’s reflex, immediately after 10 min of DF on the gastrocnemius, the soleus and the Achilles tendon [28]. Veszely et al., with a similar methodology, found similar results but also the maintenance of the changes at the 30 min follow-up [29]. It has been stated that if the experiment was performed with cutaneous anesthesia of the treated area, the behavior of the Hoffmann’s and deep tendon reflexes did not change, thus it seems that cutaneous and aponeurotic afferents did not participate in the neural answer. Moreover, the difference in behavior of reflex responses might be due more to a change in compliance of the myofascia-tendinous structure transmitting tendon knock than to a change in neuromuscular spindle sensitivity [30].

López-de-Celis et al. evaluated the immediate effects and the effects at the 30 min follow-up of a single session of DF on the neuromuscular properties of gastrocnemius in healthy subjects. A single 10 min session of DF was applied to the same regions as in the previous studies and was implemented only to one of the lower extremities. The other extremity acted as a control. After treatment, treated gastrocnemius were less rigid, showed a more relaxed state, and its contraction velocity was diminished. No differences between limbs were observed at follow-up [31].

Leite et al. [32], conducted research to study the changes in neuromuscular properties of lateral gastrocnemius immediately after a single 10 min session of DF in one group or sham DF in the other. The DF group generated a higher force and higher degree of muscle activation, measured with electromyography after DF [32].

## 4. Current Status of the Evidence about the Application of DF in Musculoskeletal and Neural Clinical Entities

Recently, a number of randomized controlled trials and experimental studies have been performed to highlight the clinical effects of DF in different clinical entities. No adverse events were reported in any study. All of the studies were subject to a risk of performance bias due to the fact that the researcher that implemented the DF, placebo or control intervention had not had the possibility to be blinded.

### 4.1. Pain in the Shoulder Region

In 2011, Barra et al. performed a double-blind randomized controlled trial in patients with chronic shoulder pain treated with DF or placebo DF. A single session of about 15 min long was performed in the muscles from the scapula, the lateral region of the shoulder and arm and the front part of the shoulder and chest. Immediately after the intervention, the active range of motion of shoulder flexion, abduction and external rotation in the group treated with the real technique were better than in the placebo group. No differences were found in pain intensity, extension or external rotation. The main limitation of the study was the variety of clinical characteristics of the sample. Chronic shoulder pain is an imprecise diagnostic, thus the possible different answers to the DF treatment in the different subgroups of patients remains unclear [33].

In 2013, Barra et al. completed the previous study, with a new study in the shoulder region, this time in patients with clinically diagnosed subacromial impingement syndrome, based on Neer and Kennedy impingement tests. The double-blind randomized controlled trial included three groups: FD, placebo FD and control. The intervention with FD or placebo FD was implemented for three weeks, with two sessions per week, non-consecutively. The three groups also received the usual treatment based on therapeutic exercises, analgesic electrotherapy and cryotherapy five days a week during the three weeks. Measurements were taken before and after treatment and at the three-month follow-up. The intervention group after treatment had better active extension and external rotation and reported better functionality than the control group. The placebo group showed better results than the control group only in extension. A higher proportion of patients in the DF group reported a feeling of clinical improvement after treatment. No differences between groups were found at the three-month follow-up. The main limitation of the results of this study was that at three months, 20% of the participants were lost. The last observations carried forward was used to replace the missing values and this conservative approach may have restricted the detection of statistically significant differences at the final assessment [10].

Thus, it seems that DF could be used to accelerate the rehabilitation process in subacromial impingement syndrome in the shoulder region. The improvements in functionality in the short term achieved with the technique might favor the earlier normalization of the patient’s daily living activities.

### 4.2. Chronic Lateral Epicondylalgia

In 2018, López-de-Celis et al. published a double-blind randomized controlled trial with three groups: intervention with real DF, intervention with sham DF and control. The purpose was to study the effects of DF on chronic lateral epicondylalgia, defined as pain in the epicondyle region for more than three months and increased pain with gripping or pressing and with the contraction or elongation of the extensor carpi radialis brevis. The intervention period was 3 weeks, and measurements were performed at baseline, at the end of the intervention and at the 3-month follow-up. The three groups received the habitual treatment, based on ultrasound, transcutaneous electrical nerve stimulation and muscular stretching, 5 days per week. Real or sham DF were implemented two times per week, non-consecutively, for around 10 min, in the lateral region of the arm, anterior and posterior forearm and epicondyle. At the end of the treatment, the DF group had better general functionality than the control and placebo groups and less pain with more pain-free grip strength, general functionality and occupational functionality than the sham group. In the follow-up, the DF group had higher pain-free grip strength than the others groups, and better general functionality than the control group. No differences between the control and placebo groups were detected at any time. A higher proportion of patients in the DF group reported a feeling of clinical improvement after treatment and at follow-up. The principal limitation of this study was the short period of follow-up, which did not allow the potential frequent recurrence of the pathology to register [8].

Thus, DF may be recommended along with standard care for the management of chronic lateral epicondylalgia.

### 4.3. Chronic Patellofemoral Pain Syndrome

Fanlo et al. studied the effects of DF on patients with chronic patellofemoral pain syndrome, which is identified as the presence of patellar pain during at least two of these activities: prolonged sitting, stair climbing, squatting, running, kneeling or jumping. Patients were treated during one week, with three 30 min sessions of DF on non-consecutive days. Anterior and lateral myofascias of the thigh were treated together with the soft tissues around the patellofemoral joint. Measurements were taken at the baseline, at the end of the treatment and after a one-week follow-up. The patella-lateral condyle distance augmented from baseline to the end of the treatment period, without changes at follow-up. Pain and functionality improved after the treatment and at follow-up [34]. Pressure pain threshold from the anterior knee joint, tibialis anterior and contralateral epicondyle of the humerus (selected as a remote point to measure widespread hyperalgesia) diminished after treatment and at follow-up. Muscle length from the rectus femoris, hamstrings and iliotibial band increased after treatment and at follow-up. A total of 97% of patients reported a feeling of clinical improvement after treatment and at follow-up. The most important limitation of this study was the short intervention and follow-up period of one week in this pathology characterized as chronic. A potential placebo effect biasing treatment effects might not be discarded [35].

These results may encourage the use of DF for the treatment of chronic patellofemoral pain syndrome, thought medium and long-term follow-up studies are needed.

### 4.4. Carpal Tunnel Syndrome

Jiménez del Barrio et al. studied the effectiveness of DF in the treatment of patients with mild to moderate carpal tunnel syndrome, diagnosed with electroneurogram by means of a double-blind randomized controlled trial with two groups: DF intervention and sham DF intervention. The protocol consisted of five sessions of DF or sham DF of around 20 min, with an interlude of two to five days between each session. The technique was applied in the myofascias of the ventral forearm and ventral tendons and the fascia of the hand. Measurements were taken at the baseline, immediately after treatment and at the one-month follow-up. Intensity of the nocturnal pain, general functionality of the upper extremity and median nerve distal motor latency and sensory conduction velocity at the wrist were better in the DF group immediately after treatment [36]. The differences in the intensity of nocturnal pain and the general functionality of the upper extremity persisted at the one-month follow-up. Electroneurophysiological data are not available on the follow-up. The main limitation of this study was that possible changes in medication intake throughout the duration of the study were not recorded [36].

A secondary analysis of the previous study was performed with a selected subgroup of patients determined to have a result in the standard median nerve neurodynamic test characterized by achieving the last movement performed in the test, elbow extension, and with data measured only after treatment and without follow-up [37]. Researchers registered the range of motion of elbow extension (more elbow extension means less mechano-sensibility in the median nerve), symptom intensity at the end of the range of motion, the sensory conduction velocity of the median nerve at the wrist, and the symptom severity and reported functionality registered together, as the outcome obtained in the Boston Carpal Tunnel Syndrome Questionnaire. All of the variables were better after treatment in the DF group [37].

Another study from Jiménez del Barrio et al. with a similar methodology found that after treatment, the DF group, with respect to the sham group, obtained lower numbness intensity and a lower cross-sectional area of the median nerve and thickness of the transversal carpal ligament, measured with ultrasound [38]. The most important limitation of this study was that patients diagnosed with bilateral pathology were included in the same group to preserve their blinding. This may have limited the effectiveness of the randomization to avoid selection bias.

These results may suggest a role for DF in the approach of carpal tunnel syndrome, though studies on severe cases and with longer follow-up periods are needed.

### 4.5. Hamstrings Shortening

Cadellans-Arróniz et al. aimed to study the effects of DF on the neuromuscular properties of shortened hamstrings, diagnosed in athletes with an outcome from a passive knee extension test of less than 160 degrees. They performed a single-blinded randomized within participant controlled clinical trial; that is, athletes with hamstrings shortening in both lower limbs were selected. One limb, randomly selected, was treated, and the other served as the control. Measurements were taken at the baseline, immediately after treatment and at the 30 min follow-up. Intervention consisted of the implementation of one session of DF of around 10 min in the myofascial tissues from the ipsilateral lumbar and gluteal region and of the dorsal thigh of the lower limb randomly selected for intervention. Immediately after treatment, stiffness of the gluteus muscle and the stiffness and tone of the biceps femoris had diminished more in the treated limb. These previous changes did not persist at follow-up, although the biceps femoris of the treated limb had relaxed more at follow-up. Other aspects of neuromuscular properties such us contractile properties measured with tensiomyography and intensity of perceived pain with a pressure of 4 Kg/cm^2^ at the thickest point of the gluteus, biceps femoris and semitendinosus muscles did not show any difference between the treated and the control limbs [39]. Immediately after treatment, gluteus maximus maximal isometric strength improved more in the treated limb, and this change persisted at follow-up. At follow-up, hamstring length had improved more in the treated limb [40]. However, no differences between the treated and control limbs were detected in the overall flexibility of the hamstring and lower back muscles, the hamstrings’ maximal isometric strength and electrical muscle activity during jumping [40]. Jump height did not change after intervention nor at the follow-up [40]. The principal limitation of this study was that as the subjects acted as their own controls, a neural central effect affecting the control limb could not be discarded. A potential placebo effect biasing treatment effects might not be discarded either.

Thus, DF may be recommended to relax and increase the length of shortened hamstrings, though more studies with higher doses and longer periods of follow-up are needed in order to facilitate the standardization of protocols for clinical practice.

### 4.6. Temporomandibular Disorders

Leite et al. performed a sham randomized controlled trial to assess the effects of DF on temporomandibular disorders in women. The participants were included if they at least suffered from muscle dysfunction in the temporomandibular region, diagnosed and classified according to the validated Portuguese version of the Diagnostic Criteria for Temporomandibular Disorders from the International Network for Orofacial Pain and Related Disorders Methodology; however, 55% of participants also had disc or joint implications. Measurements were taken at the baseline and after the 4 weeks of the DF protocol. The protocol consisted of two 10 min DF or sham technique sessions a week in the masseter and temporal muscles, bilaterally. The women treated with DF perceived lower intensity in their usual orofacial pain evoked by mouth opening and had a higher pressure pain threshold in the right temporal muscle belly than the sham group. No differences between the groups were detected in active and asymptomatic maximal mouth opening, although a higher percentage of subjects in the DF group had values of mouth opening above the average international normal level of pain-free opening (40 mm) after treatment [41]. After treatment, a lower percentage of subjects in the DF group presented moderate severity in functional limitation related to their temporomandibular disorder, with the remaining subjects in the DF group showing low levels of severity. The main limitation of this study was the heterogeneity of the clinical manifestations of the patients. A total of 55% of the patients included suffered not only from myofascial problems, but also from disc or joint disorders. Thus, more accurate criteria, excluding patients with disorders not directly afforded with DF, might favor the clarification of the effects of DF [41].

DF might be indicated to ameliorate the clinical status of patients with temporomandibular disorders, considering the positive short-term results highlighted in the previous study.

### 4.7. Tension Type Headache

Cabanillas-Barea et al. performed a single-blinded randomized controlled trial to study the effects of adding DF to the prescribed pharmacological treatment in patients with tension-type headaches diagnosed by their physician according to the International Classification of Headache Disorders criteria. The intervention consisted of three 30 min DF sessions applied with at least two days of rest between sessions in the dorsal thoracic, scapular, cervical and occipital regions. Assessments performed by a blind evaluator were carried out before and after treatment and at a one-month follow-up. The frequency of headache and the reported impact on daily living caused by the headache ameliorated more in the DF group compared with the standard care group at the follow-up (after treatment they were not assessed). The active cervical range of motion in the three planes ameliorated more in the DF group compared with the standard care group at the end of the treatment and at the one-month follow-up. The intensity of pain perceived at the moment of the assessment ameliorated more in the DF group but only after treatment and not at follow-up [42]. The worst and the usual intensity of pain ameliorated more in the DF group at the one-month follow-up (after treatment they were not assessed) [43]. A higher percentage of subjects said that they felt better in the DF group compared with the standard care group at the end of the treatment and at the one-month follow-up [42,43]. The main limitation of this study was a follow-up period of only one month in a pathology that might be chronic. Another limitation was that possible changes in medication intake throughout the duration of the study were not recorded. A potential placebo effect biasing treatment effects might not be discarded neither.

It seems that DF may be a promising method to contribute to the management of symptoms and daily living in patients with tension-type headaches.

### 4.8. Chronic Low Back Pain

In 2019, Castilho-Alonso et al. performed a randomized controlled trial to analyze the effects of DF in patients with chronic low back pain. They established two groups. The kinesiotherapy group, acting as the control group, performed a ball kinesiotherapy protocol for 50 min, three times a week for 8 weeks. The DF group performed the same protocol and received a DF treatment on the same days in the sacral region, lumbar spine and piriformis muscle. Measurements were taken before and after the protocol. Lumbar pain-related disability was minor after treatment in the DF group. No differences between the groups were detected after treatment in trunk active range of motion in flexion, extension and side bending, in abdominal strength and in pain. The main limitation of this study was that chronic low back pain is an imprecise diagnostic, thus the possible different answers to the DF treatment in the different subgroups of patients with this diagnostic remains unclear. A potential placebo effect biasing treatment effects might not be discarded [44].

Thus, DF might be a useful technique in the normalization of daily living activities in patients with low back pain.

### 4.9. Scar Treatment

In 2019, Aparecida et al. published an experimental study including 26 women with a previous cesarean surgery and some type of musculoskeletal pain somewhere. The scar was treated one time and the subjects were reevaluated 24 h later. Reductions in general pain and pain on movement, and improvements in pressure pain threshold in the vertebral spinous process, the level corresponding to the dermatome of the patient’s symptom region, and general flexibility were observed. The thermography, measured using a thermography camera in the location of the symptoms, did not change. The main limitation of this study was the absence of a control group. Another important limitation was the unspecified assessment performed [45].

The results seem promising, but the methodology of the study does not allow us to make a recommendation.

## 5. Conclusions

Current evidence primarily supports a mechanical effect of DF on connective tissue-based soft tissues. Diminished deep tendon reflex and rigidity have been registered after the implementation of DF in healthy subjects. An increase in muscle stiffness has been related to a higher risk of injury, thus, DF might be an adequate technique to prevent and treat muscular injuries.

Though there is still much to uncover, scientific evidence supports the use of the technique for the clinical approach of subacromial impingement syndrome, chronic lateral epicondylalgia, chronic patellofemoral pain syndrome, mild to moderate carpal tunnel syndrome, hamstrings shortening, temporomandibular disorders, tension-type headache and chronic low back pain.

DF could be used to accelerate the rehabilitation process in subacromial impingement syndrome in the shoulder region. The immediate improvements in functionality achieved with the technique might favor the earlier normalization of the patient’s daily living activities.

DF may be recommended to ameliorate pain-free grip strength and functionality in chronic lateral epicondylalgia in the medium term.

DF may be prescribed for the treatment of chronic patellofemoral pain syndrome due to its favorable effects on patella position, pain, functionality and muscle length from the rectus femoris, hamstrings and iliotibial band in the short term.

DF may be suggested for the alleviation of symptoms and the improvement of functionality in patients with mild to moderate carpal tunnel syndrome in the short term.

DF may be prescribed to relax and to increase the length of shortened hamstrings.

DF might be recommended for the treatment of temporomandibular disorders in women due to its favorable, immediate effects on pain, mouth opening and functionality.

DF may be prescribed for tension-type headache to ameliorate the intensity of pain, the frequency of headaches, the reported impact on daily living caused by the headache and the active cervical range of motion in the three planes in the short term.

DF might be suggested for the treatment of chronic low back pain due to its favorable, immediate effects on lumbar pain related disability.

The patient perspective about the DF treatment was always a feeling of clinical improvement when it has been registered.

Future research must afford the inclusion of more specific subgroups of patients in studies from different clinical entities. Moreover, future research should try to implement longer periods of treatment and follow-up, in accordance with the natural history of the pathologies, to establish the most suitable dosage and the effects on the progression of the pathologies. In vitro studies might be developed in the future to highlight the mechanisms of action of DF.

Additional data are essential for better recommendations in the clinical practice of DF.

## Data Availability

No new data were created or analyzed in this study. Data sharing is not applicable to this article.
